# Novel Low-Voltage Electro-Ejaculation Approach for Sperm Collection from Zoo Captive Lanyu Miniature Pigs (*Sus barbatus sumatranus*)

**DOI:** 10.3390/ani10101825

**Published:** 2020-10-07

**Authors:** Yu-Hsin Chen, Jane-Fang Yu, Yu-Jia Chang, Shih-Chien Chin, Lih-Chiann Wang, Hsiu-Lien Lin, Pei-Shiue Tsai

**Affiliations:** 1 Livestock Research Institute—Xinhua Branch, Council of Agriculture, Executive Yuan, Tainan 71246, Taiwan; yhchen@mail.tlri.gov.tw (Y.-H.C.); hllin@mail.tlri.gov.tw (H.-L.L.); 2 Department of Animal Science, National Chiayi University, Chiayi 60004, Taiwan; 3 Conservation and Research Center, Taipei Zoo, 30 Xinguang Road, Section 2, Wenshan, Taipei 11656, Taiwan; sux02@zoo.gov.tw (J.-F.Y.); r07629019@ntu.edu.tw (Y.-J.C.); sux01@zoo.gov.tw (S.-C.C.); 4 Graduate Institute of Veterinary Medicine, National Taiwan University, Taipei 10617, Taiwan; 5 Department of Veterinary Medicine, National Taiwan University, Taipei 10617, Taiwan; lcwang@ntu.edu.tw; 6 Research Center for Developmental Biology and Regenerative Medicine, National Taiwan University, Taipei 10617, Taiwan

**Keywords:** electro-ejaculation, semen collection, zoo, wild boar

## Abstract

**Simple Summary:**

Preservation of genetic materials from zoo animals is important to improve genetic diversity and to prolong the existence of endangered species for future retrieval. Methods to acquire sperm samples from the animals can be achieved by hand penile massage or by rectal stimulation with electric probe. Traditional electrical stimulation applies relatively high voltage to achieve sufficient stimulation; however, this method causes stress and discomfort to the animal. In the current study, we establish a low-voltage base electrical stimulation approach to obtain semen sample from zoo captive lanyu miniature pigs. We showed that, with our method, a sufficient amount of semen ejaculates with good sperm quality can be obtained. Future improvement on cryopreservation protocol will allow better preservation outcome for successful fertilization in this species.

**Abstract:**

Semen collection can be achieved via hand penile massage or rectal stimulation using electro-ejaculation methods. Traditional electro-ejaculation procedure applied relatively high voltage of 3–15 volts with a maximum current of 900 mA. However, these manipulations often result in great stress and discomforts in animals. In this study, we showed low-voltage electro-ejaculation procedure using 2–3 volts with a maximum current of 500 mA can efficiently stimulated ejaculations in zoo captive lanyu miniature pigs with a high success rate of 81.3% (13/16). Besides normal semen properties (semen volume, pH, sperm concentration), we demonstrated that low-voltage electro-ejaculation caused less stress in the animals, and sperm cells obtained via low-voltage electro-ejaculation exhibit low abnormality (10.3%), high viability (84.3%), motility (75.7%), progressive motility (63.7%), and acrosome integrity (88%). However, cryopreservation protocol used in the current study requires further optimization, as sperm mitochondrial function was partially compromised during freezing procedures. Taken together, we demonstrated in this study that a low-voltage electro-ejaculation approach can be used to obtain quality sperm cells from zoo captive lanyu miniature pig with less physical stress during electro-ejaculation procedure.

## 1. Introduction

Preserving genetic materials from both wildlife and zoo captive species is critical to improve genetic diversity and to prolong the existence of endangered species for future retrieval [1]. Preservation of gametes requires collection of sperm cells and/or oocytes using optimized protocols to minimize potential damages on gametes [2,3]. However, due to species-specific and individual variations, the collection, preservation, and retrieval of gametes are always challenging for veterinarians [1,4,5,6,7,8,9,10]. Semen collection in animals can be achieved by either electro-ejaculation (EEJ) or by penile hand massage. We showed in our earlier publication that some zoo captive species (e.g., chimpanzee, orangutan) can be trained for semen collection via penile hand massage using an artificial vagina [11]; however, penile hand massage for semen collection requires laborious training procedures and is a time and effort consuming process with no guarantee for success. In contrast, EEJ is a relatively fast and easy approach for semen collection as a daily practice; however, traditional EEJ protocol exhibits a greater risk to animals due to the need for deep anesthetization and high electric voltage to induce ejaculation. Earlier studies also indicated that upon EEJ, the high voltages used for stimulating ejaculation exhibited detrimental effects on sperm cells and will lead to generally suboptimal semen quality [12,13]; therefore, a modified EEJ procedure is needed to ensure animal safety and the quality of collected sperm cells.

Depending on the age at collection, and the protocols used for cryopreservation and retrieval, the quality and the survival rate of the collected gametes varies. Considering the within-herds inbreeding situation for most zoo captive species, reproduction success and the quality of the gametes are often suboptimal; therefore, for medical, husbandry, and research purposes, and to improve fertilization success, Taipei Zoo initiated a semen collection trail using a low-voltage electro-ejaculation method in order to minimize damage on sperm cells and to decrease the risks upon semen collection in zoo captive animals. Lanyu miniature pigs (*Sus barbatus sumatranus*) is a subspecies of Eurasian wild boar and is endemic to Taiwan; however, due to increasing interbreeding of lanyu miniature pigs with domestic pigs, the number of individuals with pure genetic background has gradually decreased in recent years [14,15]. To protect this unique species, Taipei zoo lunched a special conservation and breeding program. Despite some success, optimization on collecting and preserving upmost quality spermatozoa from purebred lanyu miniature pigs is still urgently needed. A fully-grown lanyu miniature pig grows mostly to a maximum size of 30–50 cm tall, and weighs around 20–50 kg [16]. Due to its size and being easy to handle, miniature pigs were being used extensively in biomedical researches, including in toxicological study [17], heart research [18], and neurodegenerative disorder studies [19]. Therefore, establishing a standard and optimized semen collection protocol is needed to improve the breeding success of lanyu miniature pigs. One critical factor for successful sperm cryopreservation is the initial quality of sperm cells at semen collection; however, adult male lanyu pigs are difficult to be trained for semen collection using either artificial vagina or penile hand massage due to their natural wild behaviors; therefore, EEJ would be particularly useful for collecting semen samples from this species on a daily basis. However, traditional EEJ is thought to cause significant stress and pain to the animals due to relatively high voltage (2–10 volts) used. The level of stress and pain during traditional EEJ procedure are evidenced by the increased vocalization, the elevated blood pressure and heart and respiratory rates during semen collection procedure [20]. Therefore, in this study, our prioritized aim is to establish a novel low-voltage EEJ semen collection protocol for zoo captive lanyu miniature pigs. Secondly, we also aim to evaluate the effects of this new protocol on frozen-thawed sperm, and to optimize cryopreservation procedure for zoo captive lanyu miniature pigs.

## 2. Materials and Methods

### 2.1. Chemicals, Reagents, Antibodies

Chemicals and reagents were obtained from Sigma-Aldrich (St. Louis, MO, USA) unless otherwise stated. Sperm incubation medium (Modified Ham’s F10, Cat.#99109) and frozen medium (Cat.#90129) used for cryopreservation experiments were acquired from Irvine Scientific (CA, USA).

### 2.2. Animals

**Figure 1:**
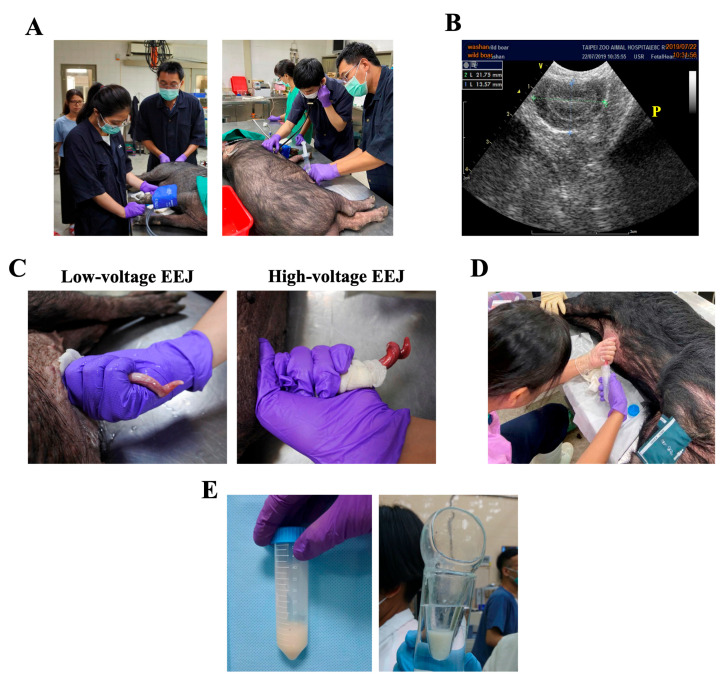
Electro-ejaculation procedures. (**A**) Anesthesia, physical parameters monitoring and preputial cavity disinfection of lanyu miniature pigs prior to electro-stimulation. (**B**) To ensure efficient stimulation, ultrasonography was used to localize electric probe at the prostate (indicated as “P”). (**C**) Appearance of protruded penis under both low-voltage EEJ (left panel) and high-voltage-EEJ (left panel) showed reddish and hyeremia penis at higher voltage stimulation. (**D**,**E**) Collection of semen ejaculates with either 50 mL sterilized plastic tube or a pre-warmed glasses container.

A total of 15 2–3-year-old domestic lanyu miniature pigs captivated in Livestock Research Institute, Council of Agriculture, Taiwan, were randomly assigned into two groups (seven for traditional high-voltage EEJ, and eight for low-voltage EEJ). Unlike conventional LYD (mixed breed of Landrace-Yorkshire-Duroc) pigs, lanyu miniature pigs exhibited brown to black appearance with relatively long and thick hair covering their body surface (Figure 1A). The body weight of lanyu miniature pigs used in this study was ranged from 30 to 75 kg. Animal handling and the acquisition of animal materials (i.e., semen ejaculates) were followed by the regulation and approval of animal welfare committee at the Taipei Zoo (protocol #10803) and at the Livestock Research Institute, Council of Agriculture, Taiwan (protocol #LRIIACUC 108-4), and all procedures were monitored and performed under the guidance of certified veterinarians.

### 2.3. Low-Voltage Electro-Ejaculation Protocol for Semen Collecting

The initial induction of the anesthesia and the maintenance of anesthesia were carried out by blow dart muscle injection of Zoletil 3–4.5 mg/kg, and 2–3% isoflurane, respectively. Body temperature, and cardiac and respiratory frequency were monitored continuously throughout the procedure (Figure 1A). EEJ normally leads to blood pressure elevation; therefore, the blood pressure of the animals was monitored with human sphygmomanometer throughout the operation (Figure 1A, left panel), and the operation would be suspended when the blood pressure was higher than 200 mmHg. Rectal feces were manually removed, and the preputial cavity was disinfected before EEJ by 0.7% iodide solution and normal saline (Figure 1A, right panel). In short, a sterilized plastic catheter was inserted into the preputial cavity after the animal was anesthetized. Preputial cavity was cleaned by three times injection of 5 mL sterilized and pre-warmed normal saline followed by 3 min gentle massage. 0.7% iodide wash solution (5 mL each, three times) was used subsequently to disinfect preputial cavity followed by additional three washes with 5 mL sterilized and pre-warmed normal saline (Figure 1A, right panel).

Ultrasonography was applied to locate the position of electric rectal probe (Figure 1B, “P” as prostate gland). Insertion of electric rectal probe at/around prostate gland normally give rise to successful and sufficient stimulation for EEJ, to standardize our operation procedure, the distance between anus and prostate gland was measured accordingly. For EEJ in lanyu miniature pigs, three -linear electrode (0.4 cm wide and 5 cm long) rectal probe with a diameter of 1.8 cm and the length of 30 cm was used.

**Figure 2:**
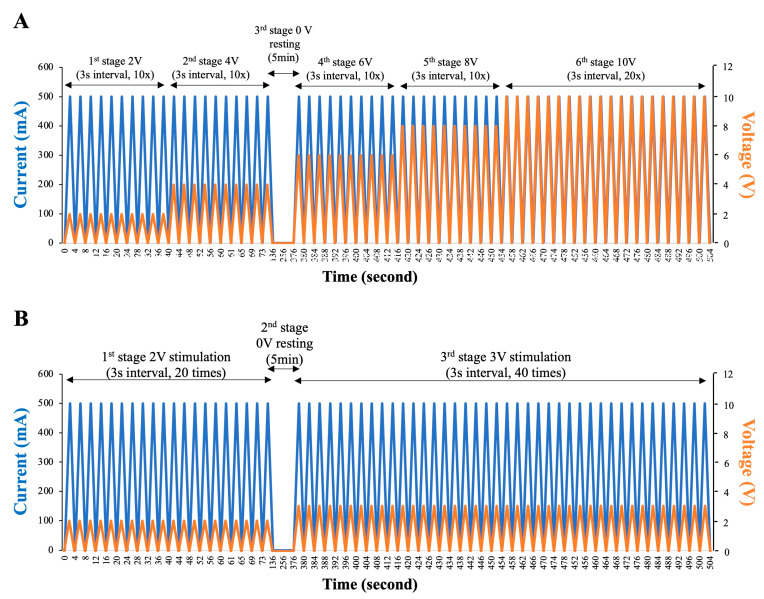
Electro-ejaculation protocols. (**A**) Traditional high voltage based stimulation protocol consisted of 6 different stages with stepwise increase of AC voltage from 2 to 10 volts (orange line) under a constant current of 500 mA (blue line). The probe was activated for 3 s for each stimulus from 2 volts for 10 stimuli, then changed to 4, 6, 8 volts for additional 10 stimuli, a final 20 stimuli with 10 volts was used. (**B**) Low-voltage based stimulation protocol established in this study consisted of 3 stages with a 2-step increase of AC voltage from 2 to 3 volts (orange line) under a constant current of 500 mA (blue line).

The electric probe covered with lubricant jelly was inserted into the rectum adjacent to the prostate gland under the guidance of ultrasound. EEJ procedures and stimulation protocols for both approaches were summarized in Figure 2. For traditional high-voltage EEJ, a constant current of 500 mA was delivered, and a gradual increase of AC voltage from 2 to maximum 10 volts was applied. Electrical stimuli were applied in a steadily increasing manner by a sine-wave electronic electro-ejaculator. The probe was activated for 3 s for each stimulus, a step-wise increase in voltage (2, 4, 6, 8, volts) was applied, and 10 stimuli for each voltage was carried out before a final 20 stimuli with 10 volts was used (Figure 2A). For low-voltage EEJ, the probe was set to deliver an AC voltage of 2–3 volts with the wave frequency of 60 Hz and an electric current of 500 mA. Electrical stimuli were also applied in a steadily increasing manner as showed in Figure 2. To minimize the stress and discomfort to the animal, the probe was activated for 3 s for each stimulus from 2 volts for 20 stimuli, then changed to 3 volts for another 40 stimuli (Figure 2B). For both approaches, each individual experienced two attempts (in total 14 attempts for high-voltage EEJ and 16 attempts for low-voltage EEJ) with a 5 min resting interval between each attempt. After stimulation, preputial cavity was squeezed to allow semen flow into a 35 °C pre-warm glasses container or a 50 mL sterilized plastic tube (Figure 1D). The presence of sperm cells was evaluated immediately under the microscopy. The color of the semen varied from light opaque to milky white (Figure 1E), the volume, the sperm concentration, and other general sperm parameters (e.g., motility, forward progressive motility, viability, acrosome integrity, and structural morphology) were also evaluated.

### 2.4. Semen Preparation and Sperm Analyses

Semen samples were evaluated immediately after the removal of gelatin content through the sterilized filter (40 µm pore size). As one of our aims was to establish an optimized cryopreservation protocol for zoo captive lanyu miniature pigs, the cryopreservation protocols described in our earlier publication for chimpanzee were applied with modifications [11]. In short, semen samples were diluted (1:1 ratio) with Beltsville thawing solution (BTS, 37.0 g/L glucose, 1.25 g/L EDTA, 6.0 g/L sodium citrate, 1.25 g/L sodium bicarbonate, and 0.75 g/L potassium chloride), and kept at 15°C for 2 h. Chilled spermatozoa were subsequently spun down (800 G, 10 min) and resuspended with the test yolk-containing frozen medium (Irvine Scientific) to achieve a final concentration of 5–10 × 10^8^ spermatozoa/mL. Sperm-test yolk mixture was cooled down at 5 °C for 2 h, chilled sperm samples were mixed (1:1 ratio) with 6% glycerol (diluted in test yolk) to reach a final concentration of 3% glycerol, before loading into plastic straws (0.25 mL) at 4 °C. Chilled straws were subsequently exposed to nitrogen vapor (4 cm over the surface of liquid nitrogen) for 10 min before being immersed and stored in liquid nitrogen. General semen and sperm quality regarding semen concentration, sperm motility, mortality, and morphological abnormality were manually evaluated by experienced and trained personnel. For morphological evaluation, standard Papanicolaou (PAP) and POPE’s staining procedures were carried out as previously described [11,21]. One hundred sperm cells were evaluated for each individual experiment, and data was expressed in percentages (%).

Propidium iodide (PI) stain was performed to evaluate sperm viability [11]. In brief, sperm cells were resuspended in Ham’s F-10 medium, and 0.5 µL of stock PI (1 mg/mL) was added to obtain a final concentration of 5 µg/mL. Samples were incubated at 37 °C in a dark room for 8 min before manual assessment under a fluorescent microscopy. Motility-related sperm parameters were analyzed using an UltiMate computer assisted sperm analysis system (CASA, Hamilton Thorne Inc., Beverly, MA, USA) as described by Broekhuijse et al. [22]. Default parameter settings were followed by recommendation of Hamilton Thorne Inc. for porcine spermatozoa. Image capture was set to 60 frames/s, a total of 45 frames were recorded per examination field. Fresh or cryopreserved spermatozoa were resuspended in pre-warmed Ham’s F10 medium, and 3 µL sperm suspension was loaded onto a standardized Leja 4-chamber counting slide (Leja Products B.V., Nieuw Vennep, The Netherlands). By the use of automated stage, five microscopic fields were analyzed within each chamber (a total of 225 frames were taken per experimental sample). At least three technical repeats were performed for each sample, and mean values and standard deviation (SD) were calculated accordingly. Motility related parameters including motility (%) and progressive motility (%, defined as VAP ≥ 25 µm/s, STR ≥ 30%) were measured and analyzed.

### 2.5. Indirect Immunofluorescent (IFA) Assays for Acrosome Integrity

Indirect immunofluorescent staining was carried out as described earlier to evaluate sperm acrosome integrity [11]. Briefly, fixed spermatozoa were washed and resuspended in PBS. A total of 20 µL of sperm suspension was added onto Superfrost Plus microscope slides (Thermo Scientific/Invitrogen), spermatozoa were allowed to attach to the slide for 10 min. Unbound sperm cells were removed with PBS, and antibody incubation was carried out immediately using *peanut agglutinin* (PNA) conjugated with Alexa 488 at a concentration of 100 μg/mL for 1 h at RT. Nuclei were stained with 4’,6-diamidino-2-phenylindole (DAPI) (Vectashield H-1200, Vector Laboratories, CA, USA) and slides were sealed with nail polish. At least 200 spermatozoa were counted in each sample under the Olympus IX83 epifluorescent microscopy (Olympus, Tokyo, JP) and analyzed with Cellsens software (Olympus).

### 2.6. Sperm Membrane Potential Evaluation by Flow Cytometry

Mitochondrial function is one of the important indicators for sperm quality. We therefore applied a membrane-permeant JC-1 dye which is commonly used to monitor mitochondrial health [23,24] to evaluate the effects of low-voltage EEJ on sperm health and to assess the effects of cryopreservation protocol on spermatozoa. Since JC-1 dye exhibited potential-dependent accumulation in the mitochondria, depolarization of mitochondria membrane potential can therefore be detected by a decrease in the red (590 nm)/green (529 nm) fluorescence intensity ratio. In this study, we stained both fresh or cryopreserved porcine sperm with JC-1, followed by a standard protocol suggested by the company, and subsequently evaluated by flow cytometry.

### 2.7. Statistical Analyses

Results were expressed as mean ± standard deviation (SD). Comparative studies of means were performed with one-way analysis of variance (ANOVA) followed by a Mann–Whitney U test. Statistical significance was set with *p* < 0.05 and was marked with an asterisk accordingly.

## 3. Results

### 3.1. Physiological Parameters of Lanyu Miniature Pig during Traditional High-Voltage and Novel Low-Voltage EEJ

**Figure 3:**
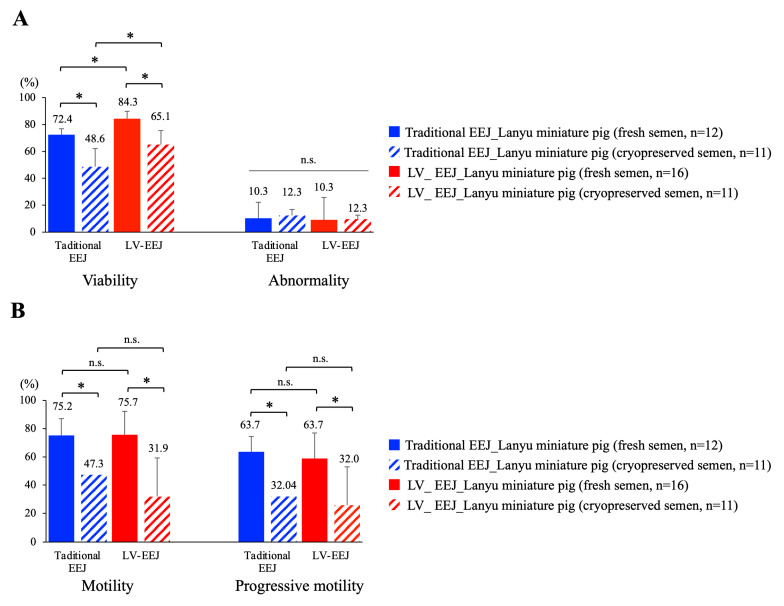
Sperm characterization (I). (**A**) Sperm viability and abnormality were evaluated manually by propidium iodide (PI) staining and standard Papanicolaou (PAP) and POPE’s staining. Clear sperm morphology can be visualized and evaluated under conventional light microscopy; more than 100 sperm cells were examined in each experimental collection. (**B**) Sperm motility and progressive motility were evaluated by computer assisted sperm analyzer (CASA). Statistical difference was set at *p* < 0.05 and significance was marked with asterisks (*), n.s. = no significant. Data presented as mean ± standard deviation (SD).

In total, 25 ejaculates from 15 miniature pigs were obtained and analyzed in this study. The size of the testes was ranged from 9.1 to 13.4 cm, and the average distance between anus and prostate gland was 15.4 ± 1.3 cm. Therefore, when inserted the electro-rectal probe for stimulation, the average insertion depth in lanyu miniature pigs was 19.4 ± 1.6 cm (Figure 3). The erection and ejaculation can be observed when applied voltage was higher than 3 volts. One particular note was that compared with low-voltage EEJ, when traditional high-voltage EEJ was applied, a relatively red and hyperemia of the protruded penis was observed (Figure 1C). We also observed that semen ejaculates became gelatinous when a higher stimulatory voltage was applied (>3 volts, data not showed). Based on data presented in Figure 3, core body temperature was not different between the two EEJ protocols; other parameters evaluated, i.e. respiratory rate, heart rate, and systolic blood pressure were all significantly elevated post-EEJ when high voltage was applied (Figure 3B). These changes were not detected when low-voltage EEJ was applied. For example, blood pressure was maintained mostly under 120 mmHg and no significant changes was detected between pre- and post EEJ (106.5 vs. 102.8 mmHg for pre- vs. post EEJ blood pressure), (Figure 3B). More importantly, when compared with traditional high-voltage EEJ of which 12.7 ± 4.1 mL semen with sperm concentration of 2.11 × 10^6^ sperm/mL was collected, an average amount of 13.1 ± 7.6 mL semen with sperm concentration of 1.57 × 10^6^ sperm/mL can be collected via low-voltage EEJ (no differences between two collection protocols) indicated sufficient stimulation was achieved using low-voltage stimulation protocol (Table 1). Based on the number of attempts and the amount of semen collected, we showed that this new low-voltage EEJ approach had a high success rate of 81.3% (13/16) on the collection of semen ejaculates with considerable amount of semen quantity (Figure 1E, Table 1), which is similar to that of traditional high-voltage EEJ protocol (85.7% success rate. 12/14).

### 3.2. Sperm Viability, Morphology, and Motility Were Maintained with Low Voltage EEJ

When sperm viability, abnormality, motility, and progressive motility were evaluated and compared, we observed that sperm samples obtained from low-voltage EEJ have a significantly higher viability of 84% (closed red bar) when compared with traditional EEJ (72%, closed blue bar) with relatively low abnormality rate (~10%) prior to cryopreservation. In contrast to traditional EEJ, of which less than 50% of the sperm cells (48.6%) remained alive after cryopreservation, a relatively higher percentage of sperm cells (65.1%) remained alive after cryopreservation procedure when low-voltage EEJ was used for semen collection (Figure 3A). For sperm motility, we measured 75.7% sperm motility with 63.7% progressive motility for lanyu miniature pigs (Figure 3B) prior to cryopreservation. Moreover, when compared with traditional EEJ, no differences can be detected between two stimulation approaches, indicated low-voltage EEJ did not affect sperm motility. However, when cryopreserved sperm cells were analyzed, we observed a drastic decline in sperm motility and progressive motility (>50% fold decrease) from both collection protocols, which suggested that optimization of cryopreservation protocol is needed (Figure 3B).

### 3.3. Sperm Acrosome Integrity Was Maintained, but Mitochondria Membrane Potential Was Compromised

**Figure 4:**
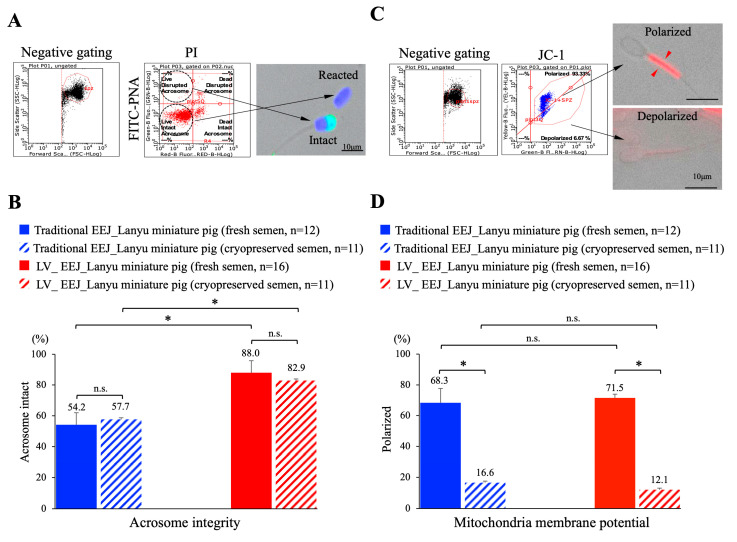
Sperm characterization (II). (**A**) Sperm acrosome integrity in live sperm was evaluated by co-staining FITC-PNA (for acrosome integrity) and propidium iodide (PI). Gating of sperm and non-sperm cells was first performed, and events counted in the second (live, acrosome disrupted) and the third (live, acrosome intact) quadrant were presented and compared. (**B**) Quantitative analysis of acrosome integrity in zoo captive lanyu mini pigs before and after cryopreservation showed significant difference regarding acrosome integrity between traditional and low-voltage EEJ indicated low-voltage EEJ caused less damages on acrosome structure. (**C**) Sperm mitochondria membrane potential was evaluated by JC-1 stain. Gating of sperm and non-sperm cells was first performed and illustration of polarized (healthy) and depolarized (unhealthy) sperm were shown. (**D**) Quantitative analysis regarding mitochondria membrane potential showed no differences between two stimulation protocols (between solid closed bars), indicated low-voltage EEJ did not compromise mitochondria function as compared with traditional EEJ. Significant declined in percentage of sperm cells with polarized mitochondria membrane potential was detected after cryopreservation in both protocols. Statistical difference was set at *p* < 0.05 and significance was marked with asterisks (*), n.s. = no significant. Data presented as mean ± standard deviation (SD). In total 10,000 events were counted in flow cytometry analysis.

Acrosome is the most important cellular compartment of a sperm cell; premature, belated, or defective acrosome reaction will lead to infertility. When sperm acrosome integrity was assessed by lectin PNA (Figure 4A), we observed that sperm cells collected via low-voltage EEJ had significantly higher percentage of acrosome intact sperm (88.0% and 82.9% for fresh and cryopreserved samples, respectively) which was similar to the percentage in LYD breed. However, when traditional EEJ was applied, a much lower percentage of acrosome intact sperm was counted (54.2% and 57.7% for fresh and cryopreserved samples) (Figure 4B). These data indicated that current cryopreservation protocol did not lead to significant damage on sperm acrosome structure, but traditional high-voltage EEJ caused more damages on sperm acrosome integrity than low-voltage EEJ. Strikingly, when mitochondria membrane potential was assessed by specific marker JC-1 (Figure 4C), we detected in fresh sperm samples that the majority of the sperm cells exhibited polarized membrane potential (68.3% and 71.5% for traditional and low-voltage EEJ, respectively); however, a sharp decline in mitochondria membrane potential was measured in cryopreserved sperm samples for both protocols (16.6% and 12.1% for traditional and low-voltage EEJ, respectively), suggesting that mitochondria function was severely compromised during current cryopreservation procedures (Figure 4D).

## 4. Discussion

Reproduction and gamete cryopreservation in wild or zoo captive animals are always challenging issues due to the limited number of individuals, social hierarchical structure, or insufficient background knowledge regarding species-specific variations. These yet to be resolved barriers often result in aging of the animals and suboptimal gamete quality, which subsequently increase genetic defect of the embryos and decreases reproductive success. The key for successful assisted reproduction (e.g., artificial insemination, in vitro fertilization) or cryopreservation is the quality of the gametes as collected spermatozoa or oocytes will undergo a series of manipulations prior to insemination back to the female. Semen collection can be achieved by various approaches, including hand penile massage [11], elector-ejaculation [20], or by the use of artificial surrogates. Semen collection via penile massage is considered a less invasive and mostly, with positive reinforcement, rewarding approach; however, it requires laborious training of the animal [11]. On the other hand, an electrostimulation-based approach is a more reliable and routine procedure for semen collection, and can be applied on most of the species with a relatively high success rate. Traditional EEJ for semen collection uses relatively high voltage to reach sufficient stimulation; however, the application receives increasing attention and criticisms for being inhumane due to animal well-being concern [25]. This is mostly owing to animals that are exposed to traditional EEJ procedures sometimes vocalizing and struggling due to the high voltage used (0–13 volts, 15 Hz with maximum current of 900 mA), and as a result, these animals experiencing stress and pain associated with the traditional EEJ approach [20]. In the current study, we noticed that when traditional EEJ was used, besides core body temperature being stable, all the other physical parameters (i.e., respiratory rate, heart rate, and systolic blood pressure) were elevated, demonstrating that the animals under this protocol experienced significant stress. Moreover, a red and hyperemic appearance of the penis was observed when compared with a normal pink appearance when low-voltage EEJ was used. With increasing concern regarding the traditional approach using relatively high voltage for stimulation, imminent need for alternative approaches is required. We showed in the current study that low voltage of 2–3 volts can efficiently stimulate ejaculation with sufficient semen quantity (an average of 13.1 ± 7.6 mL) for cryopreservation, research, and medical purposes; more importantly, neither core body temperature, nor heart rate and blood pressure were increased during or post-EEJ procedure, indicating that the low-voltage EEJ used in this study did not significantly cause stress or pain to the animals.

Parameters commonly used for semen quality assessment consist of sperm count, semen characteristics, sperm motility (can be evaluated by CASA), mortality (viability), and morphology [26]. Earlier studies from Ohl et al. in beagles, and Brackett and Lynne in human patients, both showed that sperm cells collected via traditional EEJ exhibited lower total sperm count and lower percentage of motile sperm when compared with hand penile massage [27,28]. We showed that in the current study, sperm cells collected from low-voltage electro-stimulation exhibited low percentage of abnormality (10%) with high (75.7%) motility rate. These values were similar to the results of traditional high-voltage EEJ approach, indicating that at least the low-voltage EEJ established in the current study did not lead to detrimental effects on sperm motility and morphology.

Other important indications for sperm quality and their ability for successful fertilization are sperm acrosome integrity and sperm mitochondria membrane potential [29]. Sperm acrosome contains various hydrolytic enzymes that will be released upon acrosome reaction (AR). Successful AR facilitates sperm penetration through the zona pellucida of the oocyte [30]; however, premature or belated AR will lead to sub/infertility. For natural mating, AR reaction occurs when sperm cells get in contact with the oocyte, and is thought to drive by a gradient of chemokines released by cumulus oocyte complex (COCs) [31,32]. We showed that low-voltage EEJ did not compromise acrosome integrity, nor promote the occurrence of premature AR as both fresh and cryopreserved sperm cells maintained high percentage of intact acrosome (~82.9–88%). Apart from the above-mentioned parameters, we observed drastic decrease in sperm motility, progressive motility, and mitochondria membrane potential in post-thawed cryopreserved sperm cells. Sperm motility and progressive motility are some of most important factors to drive successful fertilization upon natural mating, as declined linear forward movement results in low fertilization rate. Movement of the sperm cells requires constant energy supplementation (e.g., ATP) provided by functional mitochondria. Defective or dysfunctional sperm mitochondria has been shown to associate with low fertility in many species, including humans, horses, and dogs [8,33,34,35,36]. We detected severe decline in sperm membrane potential after cryopreservation process, suggesting that the function of mitochondria was affected after cryopreservation. Since around 68–75% of the sperm cells maintained their mitochondria membrane potential prior to cryopreservation, we considered that the defective mitochondria membrane potential resulted from current suboptimal cryopreservation protocol and/or medium used rather than a direct effect from the semen collection procedure of low-voltage EEJ. The damaged mitochondria likely resulted in insufficient ATP production, and thereafter reflected on low sperm motility and progressive motility in cryopreserved spermatozoa. Sperm cells from fresh semen exhibited good motility and mitochondria function; this suboptimal cryopreservation outcome indicated the need for substantial adjustments on the current cryopreservation procedure. As a standardized freezing protocol has been established for Laurence-Yorkshire-Durence (LYD) crossbred boar, adjustments based on the same cryopreservation protocol should be considered.

Despite the seeming success of this novel EEJ approach, one thing we have to noted is that in this study, we obtained on average 13.1 mL semen ejaculate with a sperm concentration of 1.57 million sperm per ml (therefore, a total ~20 million sperm cells per ejaculate). This is still a relatively low quantity of semen and sperm cells when compared with a trained LYD crossbred boar (150–200 mL) in the commercial farm. As 1 billion sperm cells is considered a minimum artificial insemination dose in agricultural pigs, the value of 2% of this number acquired in this study is still unclear for the success for artificial insemination.

## 5. Conclusions

In conclusion, our study showed low-voltage electroejaculation procedure using 2–3 volts with a maximum current of 500 mA efficiently stimulated ejaculations in zoo captive lanyu miniature pigs. Moreover, low-voltage EEJ caused less stress to the animals as physical parameters, such as core body temperature, heart rate, and blood pressure were stable throughout the stimulation process. More importantly, sperm cells obtained via low-voltage EEJ exhibited normal viability, low abnormality, high motility, progressive motility, and acrosome integrity. However, current cryopreservation procedure requires substantial adjustments as severe mitochondria damage was detected in cryopreserved sperm cells.

## Figures and Tables

**Table 1:** Physiological parameters and general semen characteristics. **(A)** General physiological parameters of lanyu miniature pigs used in the study. Age of the animals, body weight, the size of the testes and the length of anus to prostate gland was measured and recorded. **(B)** Core body temperature, respiratory rate, heart rate, and systolic blood pressure were monitored throughout the experiment, pre- and post-EEJ values were compared. **(C)** Semen volume, pH, and sperm concentration were recorded for both traditional and low-voltage EEJ. Asterisks indicated statistical difference between pre- and post EEJ procedure.

**(A)**				
	**Zoo Captive Lanyu Miniature Pig (*n* = 15)**			
Age of the animals (year)	2.67 ± 0.4 (2–3)			
Weight of the animals (kg)	52.9.1 ± 12.1 (30–75)			
Length of right testis (cm)	10.5 ± 1.5 (9.1–12.5)			
Length of left testis (cm)	10.9 ± 1.1 (9.7–13.4)			
Length of anus to prostate gland (cm)	15.4 ± 1.3 (14.0–18.5)			
Depth of electro-probe insertion (cm)	19.4 ± 1.6 (14.8–22.0)			
**(B)**				
	**Traditional EEJ** **(7 Individuals, 12 Ejaculates)**		**Low-Voltage EEJ** **(8 Individuals, 13 Ejaculates)**	
Core body temperature (°C)	Pre-EEJ	Post-EEJ	Pre-EEJ	Post-EEJ
Respiratory rate ( /min)	38.8 ± 0.6	38.4 ± 0.7	38.6 ± 0.9	38.0 ± 1.1
Heart rate ( /min)	27.7 ± 4.1	34.0 ± 3.5 *	28.4 ± 3.0	29.4 ± 2.8
Systolic blood pressure (mmHg)	103.7 ± 19.1	131.4 ± 12.1 *	96.0 ± 9.1	101.9 ± 22.5
**(C)**				
	**Traditional EEJ** **(7 Individuals, 12 Ejaculates)**		**Low-Voltage EEJ** **(8 Individuals, 13 Ejaculates)**	
Semen volume (mL)	12.7 ± 4.1 (3.6–20.5)		13.1 ± 7.6 (3–32.3)	
Semen pH	8.45 ± 0.1 (8.4–8.6)		7.98 ± 0.41 (7.1–8.5)	
Semen concentration (10^6^/mL)	2.11 ± 1.87 (0.6–5.9)		1.57 ± 1.48 (0.4–6.8)	
